# Potential Whole-Cell Biosensors for Detection of Metal Using MerR Family Proteins from *Enterobacter* sp. YSU and *Stenotrophomonas maltophilia* OR02

**DOI:** 10.3390/mi12020142

**Published:** 2021-01-29

**Authors:** Georgina Baya, Stephen Muhindi, Valentine Ngendahimana, Jonathan Caguiat

**Affiliations:** 1 Department of Biological and Chemical Sciences, Youngstown State University, Youngstown, OH 44555, USA; gnbaya@student.ysu.edu; 2 Department of Biological Sciences, University of Toledo, Toledo, OH 43606, USA; stephen.muhindi@rockets.utoledo.edu; 3 Biology Department, Lone Star College-CyFair, 9191 Barker Cypress Rd, Cypress, TX 77433, USA; valentine.m.ngendahimana@lonestar.edu

**Keywords:** whole-cell biosensor, MerR family protein, ZntR, CueR, bacterial metal resistance, HgCl_2_, ZnCl_2_, CuSO_4_, HAuCl_4_·3H_2_O, *Enterobacter*, *Stenotrophomonas maltophilia*

## Abstract

Cell-based biosensors harness a cell’s ability to respond to the environment by repurposing its sensing mechanisms. MerR family proteins are activator/repressor switches that regulate the expression of bacterial metal resistance genes and have been used in metal biosensors. Upon metal binding, a conformational change switches gene expression from off to on. The genomes of the multimetal resistant bacterial strains, *Stenotrophomonas maltophilia* Oak Ridge strain 02 (*S. maltophilia* 02) and *Enterobacter* sp. YSU, were recently sequenced. Sequence analysis and gene cloning identified three mercury resistance operons and three MerR switches in these strains. Transposon mutagenesis and sequence analysis identified *Enterobacter* sp. YSU zinc and copper resistance operons, which appear to be regulated by the protein switches, ZntR and CueR, respectively. Sequence analysis and reverse transcriptase polymerase chain reaction (RT-PCR) showed that a CueR switch appears to activate a *S. maltophilia* 02 copper transport gene in the presence of CuSO_4_ and HAuCl_4_·3H_2_O. In previous studies, genetic engineering replaced metal resistance genes with the reporter genes for β-galactosidase, luciferase or the green fluorescence protein (GFP). These produce a color change of a reagent, produce light, or fluoresce in the presence of ultraviolet (UV) light, respectively. Coupling these discovered operons with reporter genes has the potential to create whole-cell biosensors for HgCl_2_, ZnCl_2_, CuSO_4_ and HAuCl_4_·3H_2_O.

## 1. Introduction

**Figure 1:**
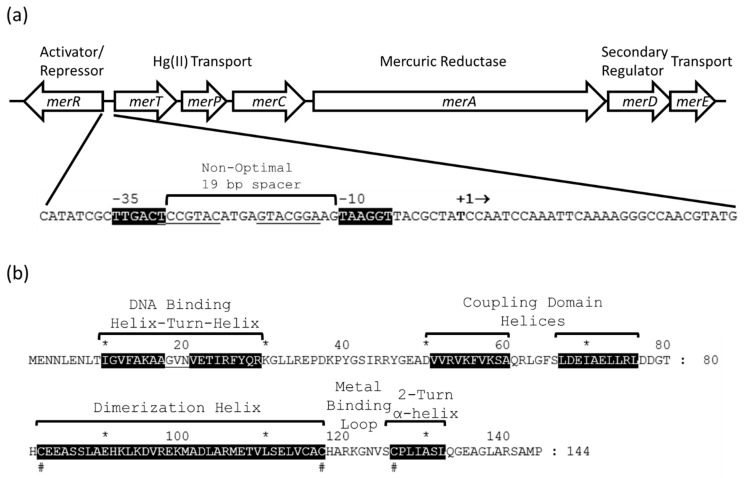
The Tn*21* mercury resistance (*mer*) operon and MerR protein activator/repressor switch. (**a**) The genes *merTPCAD* are transcribed to the right and *merR* is transcribed to the left. The MerR protein binds as a dimer to the DNA operator region (underlined) between the −10 and −35 RNA polymerase binding regions (highlighted in black). The non-optimal 19 base pair (bp) spacer between the −10 and −35 regions prevents RNA polymerase from initiating transcription efficiently. In the absence of Hg(II), MerR binding to the DNA acts as an off switch to repress transcription. In the presence of Hg(II), MerR acts as an on switch by shortening the distance between and aligning the −10 and −35 regions so that RNA polymerase can initiate transcription of the *merTPCAD* genes efficiently. Transcription begins at the nucleotide base (bold) below + in +1. The first three base pairs, CAT, on the far left marks the beginning of MerR translation. The last three base pairs, ATG, on the far right marks the beginning of MerT translation. (**b**) The Tn*21* MerR amino acid residue primary sequence. The helix-turn-helix domain binds to the operator region of the promoter in the DNA. The coupling domain links the DNA binding domain to the metal binding domain. The metal binding domain consists of the dimerization helix, metal binding loop and 2-turn α-helix. The dimerization helix links two identical MerR polypeptides to form an antiparallel coiled coil. The # below the sequences designates conserved cysteine amino acid residues which bind to Hg(II). The * and numbers above the MerR primary sequence denote the position of every 10 amino acid residues.

Whole-cell biosensors are highly anticipated in the field of on-site detection [1,2,3]. Metal-resistant bacteria control the expression of their metal resistant genes using protein switches. They activate or turn them on in the presence of toxic metals and repress them or turn them off in the absence of metal to conserve energy. The Tn*21* mercury resistance operon (*mer*) from *Shigella flexneri* has been well studied [4] and the protein switch, MerR, controls its expression [5,6]. The operon consists of the genes, *merT*, *merP*, *merC*, *merA*, *merD*, *merE* (*merTPCADE*) and *merR* (Figure 1a) [7,8]. The genes, *merTPCADE*, are transcribed in the opposite direction in relation to *merR*. MerT, MerC and MerE are cytoplasmic membrane proteins, while MerP is a periplasmic protein [9,10]. All four transport divalent mercury (Hg(II)) into the cells. Then, MerA, mercuric reductase, reduces it to elemental mercury (Hg(0)), which volatilizes from the microorganisms environment [11]. MerR is an activator/repressor protein switch [12], which binds to the operator region of the *merTPCADE* and *merR* promoters and represses the expression of *merTPCADE* and *merR* in the absence of Hg(II). When Hg(II) is present, it activates the expression of *merTPCADE*, while still repressing the expression of *merR*. Even under this repressed state, enough *merR* is transcribed and translated to produce MerR protein that efficiently regulates *merTPCADE* expression. When the concentration of mercury decreases below toxic levels, MerD restores the operon to the repressed state [13,14].

The MerR protein has three domains: a DNA binding domain, a mercury binding domain and a coupling domain (Figure 1b) [12,15,16,17]. It binds as a homodimer, two identical polypeptides, to the operator in the DNA using a helix-turn-helix motif. The binding or operator region in the DNA is located between the −10 and −35 RNA polymerase binding sites of the promoter and consists of an inverted repeat (Figure 1a). Each polypeptide in the homodimer binds to one of the repeats. The mercury binding domain contains a dimerization helix, a metal binding loop and a 2-turn alpha-helix. The dimerization helices link the dimer together in an antiparallel orientation and form a coiled coil [18]. The protein uses the sulfhydryl groups from three cysteine amino acid residues, cys82, cys117 and cys126, to bind to mercury. Since it forms a dimer, it contains two Hg(II) binding sites with a binding site consisting of cys82 and cys117 from the dimerization helix of one polypeptide and cys126 from the 2-turn α-helix of the other polypeptide. In each monomer, the coupling domain contains two alpha helices and links the mercury binding domain to the DNA binding domain. The optimal number of nucleotides between the between the −10 and −35 sites of most promoters is 17 base pairs, which allows RNA polymerase to bind to the DNA and initiate transcription [19]. The *merTPCADE* promoter contains a non-optimal 19 base pair spacer [20]. When MerR binds to Hg(II), conformational changes in the metal binding, coupling and DNA binding domains [21] shorten the distance between and aligns the −10 and −35 sites so that RNA polymerase efficiently binds to these sites and activates or switches on the expression of the resistance genes [22].

Reporter genes are useful for studying gene expression levels activated by MerR in the presence of Hg(II) [23]. The enzyme, β-galactosidase (LacZ), hydrolyzes lactose to form glucose and galactose. Ortho-nitrophenyl-β-galactoside (ONPG) is an analog of lactose. When LacZ hydrolyzes ONPG it produces a yellow color [24]. Previously, replacement of *merTPCADE* with *lacZ* as a reporter gene and the use of ONPG as a color indicator detected Hg(II) at concentrations between 0.5 and 1 ng/mL [25]. Since the Environmental Protection Agency (EPA) limit is 2 ng/mL or 10 nM, this system can serve as an effective biosensor to detect toxic levels of Hg(II) in a water sample [26].

MerR family proteins serve as activator/repressor protein switches for other metal resistances [27,28]. They each contain a DNA binding domain, a metal-binding domain, and a coupling domain with similar motifs in each domain. They also bind as dimers to inverted repeats associated with their −10 and −35 promoter regions and repress expression in the absence of metal and activate expression in the presence of metal. Some examples of MerR family proteins are ZntR, which regulates zinc resistance, and CueR which regulates copper and sometime gold resistance in a wide variety of microorganisms [17].

*Stenotrophomonas maltophilia* Oak Ridge strain 02 (*S. maltophilia* 02) and *Enterobacter* sp. YSU were isolated from a heavy metal contaminated site in Oak Ridge, TN [29]. They are both resistant to HgCl_2_, ZnCl_2_, CuSO_4_, HAuCl_4_ and other metal salts. The genomes of both strains were sequenced. Since these strains are multimetal-resistant, we expected to find genes for MerR and other MerR family proteins in these strains. Basic local alignment search tool (BLAST) analysis of the genomes using Tn*21 merR* identified two potential *merR* genes and a *cueR* gene in *S. maltophilia* 02. It also identified one potential *merR* gene, a *zntR* gene and a *cueR* gene in *Enterobacter* sp. YSU. In this article, the resistance genes that are putatively regulated by these MerR family proteins have been identified through BLAST analysis, cloning techniques and transposon mutagenesis. Replacing these resistance genes with a reporter gene makes them potentially useful as whole-cell biosensors.

## 2. Materials and Methods

### 2.1. Bacterial Strains, Plasmids and Media

*Stenotrophomonas maltophilia* Oak Ridge strain 02 (ATCC #53510) was purchased from the American Type Culture Collection (Manassas, VA, USA), *Enterobacter* sp. YSU was described previously [29] and StrataClone SoloPack Competent Cells and the plasmid, pSC-A-amp/kan, were purchased from Agilent (Santa Clara, CA, USA) as components of the StrataClone PCR (Polymerase Chain Reaction) Cloning Kit. TransforMax™ EC100D™ pir-116 *Escherichia coli* (*E. coli*) was purchased from Lucigen (Middleton, WI, USA) [30].

Lennox LB medium was purchased from Amresco (Solon, OH, USA), and R3A-tris medium was described previously [29]. When required, LB or R3A-tris medium was supplemented with 100 µg/mL ampicillin (Amresco), 50 µg/mL kanamycin (Amresco) and varying concentrations of HgCl_2_, ZnCl_2_, CdCl_2_, CuSO_4_ (Fisher Scientific, Fair Lawn, NJ, USA) and HAuCl_4_·3H_2_O (Amresco).

### 2.2. Genomic Sequencing and Sequence Analysis

A pellet from 1 mL of cells was sent to GENWIZ (South Plainfield, NJ, USA) for DNA extraction, library preparation and Next Generation Sequencing using the Illumina MiSeq 2 × 150 bp configuration with an average of ~2 Gb of raw data and a coverage of about 400× coverage per sample. The orientation and order of the resultant contigs were determined using Mauve software [31] with the *Enterobacter hormaechei* strain MS7884A chromosome (accession CP022532.1) as a reference for *Enterobacter* sp. YSU and *Stenotrophomonas maltophilia* strain NCTC13014 genome assembly (accession LR134301.1) as a reference for *S. maltophilia* 02. BLAST analysis using these same reference sequences was used to estimate the length of gaps between the contigs [32,33,34]. Because the next-generation sequencing method was not designed for plasmids, plasmid sequences for *Enterobater* sp. YSU were removed. There were no plasmid sequences for *S. maltophilia* 02. The sequences were then annotated using the National Center for Biotechnology Information (NCBI) Prokaryotic Genome Annotation Pipeline (PGAP) [35,36]. The accession numbers are CP059487 for *Enterobacter* sp. YSU and CP056088 for *S. maltophilia* 02. DNA and protein sequences were aligned using the default settings for MEGA version X software [37].

### 2.3. Polymerase Chain Reaction (PCR)

All PCR reactions were performed using 2X GoTaq^®^Green Master Mix (Promega, Madison, WI, USA). The Master Mix was diluted to 1X with nuclease free water (Amresco), 0.25 μM primer pairs (Integrated DNA Technologies, Coralville, IA, USA) and DNA template. After an initial denaturation step at 95 °C for 2 min, the reaction was incubated for 35 cycles of 95 °C for 1 min for denaturing, 55–65 °C for 1 min depending on the annealing properties of the primers, and 72 °C for 1 min/kb copied for synthesis. After a final 72 °C extension step for 10 min, the reactions were held at 10 °C. The *S. maltophilia* 02 *mer* operon 1 (S02-mer1) was copied using the primers, S02_mer1_FWD (5′ GTTCCGGCGTCGTCCATCCATC 3′) and S02_mer1_REV (5′ CACCAAGCACCAACTCGGTCCC 3′). The *S. maltophilia* 02 *mer* operon 2 (S02-mer2) was copied using the primers, S02_mer2_FWD (5′ CACAGAGCCTGCGAATCGGCAA 3′) and S02_mer2_REV (5′ CCTCGTATGGGGCAGGCTGAGA 3′). *S. maltophilia* 02 genomic DNA was used as a template for both reactions. The *Enterobacter* sp. YSU *mer* operon (YSU-mer) was copied using the primers, YSU_mer_FWD (5′ ATCGGCAAATGGCAGGGACAGG 3′) and YSU_mer_REV (5′ TACGCCGGTGACAACACATCGC 3′). This reaction used genomic DNA from *Enterobacter* sp. YSU as a template. The reactions for all three *mer* operons used 65 °C for the annealing temperature and an extension time of 7 min. The *S. maltophilia* 02 copper-translocating P-type ATPase gene and the adjacent *cueR* gene was PCR amplified using the primers, CuR-ATPase2_1 (5′ CCAACCAGATCTCCACCAA 3′) and CuR-ATPase2_2 (5′ GGTGATCAATGCCACCAAGT 3′). *S. maltophilia* 02 genomic DNA was used as a template. The annealing temperature was 60 °C, and the extension time was 4 min. Complementary DNA (cDNA) was used as a template in reverse transcriptase polymerase chain reactions (RT-PCR) with the cueR_2_FWD (5′ CTCTGGAATGACACCTCGCGGC 3′) and cueR_2_REV (5′ CCATCTGCCGCATGTGCTCGAT 3′) primers for the *cueR* gene, the ATPase_2_FWD (5′ GTGCTGGAGATGGGTTCGCACC 3′) and ATPase_2_REV (5′ GCCGGGAAGCCCTTCTGGTAGA 3′) primers for the copper translocating P-type ATPase gene and the S02_GAPDH_F (5′ AAACCGCGCAGAAGCACATCGA 3′) and S02_GAPDH_R (5′ GCCGGCGTAGGTCTTGTCGTTC 3′) primers for the glyceraldehyde-3-phosphate dehydrogenase (GAPDH) control. The annealing temperature for the ATPase gene, *cueR* and GAPDH was 57 °C, and the extension time was 30 s.

### 2.4. Transposon Mutagenesis, Cloning and DNA Purification

Zinc and copper mutants were generated by transposon mutagenesis using the EZ-Tn*5*™ <R6K*γori*/KAN-2>Tnp Transposome™ Kit (Lucigen, Middleton, WI, USA) as described previously [38,39,40], except mutants were screened using 1 mM ZnCl_2_ or 1 mM CuSO_4_ on R3A-tris medium [29] PCR DNA fragments were cloned using the StrataClone PCR Cloning Kit (Santa Clara, CA, USA). Genomic DNA was purified using the Wizard^®^ Genomic DNA Purification Kit (Promega), and plasmid DNA was purified using the Wizard^®^ Plus SV Minipreps DNA Purification System kit (Promega).

### 2.5. Minimal Inhibitory Concentrations (MICs)

Minimal inhibitory concentrations (MICs) were determined using a replica plating technique [24]. Bacterial colonies were gridded in triplicate on R3A-tris agar medium and grown overnight at 30 °C. For mercury MICS, the master plate was replica plated onto R3A-tris agar medium containing 0, 10, 20, 30, 40, 50, 60, 70, 80, 90 and 100 µM HgCl_2_. After incubating the plates at 30 °C overnight, the minimal inhibitory concentration (MIC) was determined by the lowest concentration of mercury that prevented growth. Copper MICs were determined using a similar protocol, except colonies were gridded on LB agar plates and replica plated onto LB agar medium containing 0, 0.1, 0.5, 1, 2 and 3 mM CuSO_4_. Zinc MIC’s were determined using R3A-tris agar medium for replica plating on 0, 0.2, 0.3, 0.4, 4 and 5 mM ZnCl_2_. Cadmium MICs were determined using R3A-tris agar medium for replica plating on 0, 10, 50, 100, 200 and 300 µM CdCl_2_. Gold MICs were determined by replica plating on R3A-tris agar medium containing 0, 10, 20, 30, 40 and 50 µM HAuCl_4_·3H_2_O.

### 2.6. RNA Purification, cDNA Synthesis and Reverse Transcriptase Polymerase Chain Reaction (RT-PCR)

An overnight culture of *S. maltophilia* 02 grown in LB medium was diluted 1:20 in fresh LB medium in three separate cultures and grown at 30 °C. After 1.5 h of growth, water was added to one tube as a control, HAuCl_4_·3H_2_O was added to a concentration of 200 µM to the second tube and CuSO_4_ was added to a concentration of 1 mM to the third tube. One hour after metal exposure, 100 μL of cells were added to 200 μL of RNAprotect^®^ Bacteria Reagent (Qiagen, Germantown, MD, USA), pelleted and stored at −80 °C. Total RNA from thawed cells was purified using the Qiagen RNeasy^®^ Protect Bacteria Kit according to the manufacturer’s instructions. Complementary DNA (cDNA) was prepared from 93 ng of RNA using random primers and Protoscript from NEB (Bevery, MA, USA) according to the manufacturer’s instructions. The cDNA was PCR amplified as described above, and RT-PCR reactions were separated on a 2% agarose gel.

## 3. Results and Discussion

### 3.1. Potential MerR Sensors Switches in S. maltophilia 02 and Enterobacter sp. YSU

**Figure 2:**
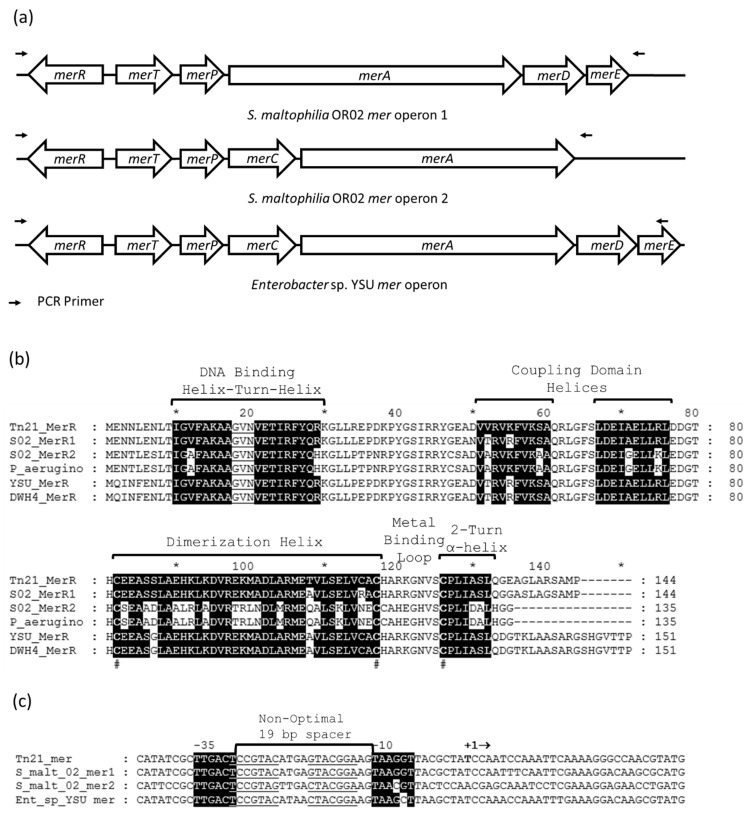
The *S. maltophilia* 02 and *Enterobacter* sp. YSU mercury resistance (*mer*) operons and MerR protein activator/repressor switches. (**a**) Mercury resistance operons in *S. maltophilia* 02 and *Enterobacter* sp. YSU. The genes between the primers were copied by polymerase chain reaction (PCR), cloned and tested for the Hg(II) resistance phenotype. (**b**) Prediction of the *S. maltophilia* 02 and *Enterobacter* sp. YSU MerR secondary structure using the Tn*21*, *Pseudomonas aeruginosa*, and *Enterobacter cloacae* plasmid DWH4 MerR amino acid residue primary sequences. Each contain a DNA binding domain, a coupling domain and a metal binding domain. The # below the sequences designates conserved cysteine amino acid residues which bind to Hg(II). The * and numbers above the MerR primary sequences denote the position of every 10 amino acid residues. (**c**) Prediction of the *S. maltophilia* 02 and *Enterobacter* sp. YSU *mer* promoter regions using the Tn*21 mer* promoter. All appear to contain non-optimal 19-bp spacers and MerR binding regions between the −10 and −35 region. Identical nucleotides in the −10 and −35 regions are highlighted in black. Transcription begins at the nucleotide base (bold) below + in +1. The first three base pairs, CAT, on the far left marks the beginning of MerR translation. The last three base pairs, ATG, on the far right marks the beginning of MerT translation.

BLAST analysis using the Tn*21* MerR amino acid residue sequence (accession P0A2Q9) against the protein databases from *S. maltophilia* 02 and *Enterobacter* sp. YSU revealed that *S. maltophilia* 02 contains two potential mercury resistance operons, and *Enterobacter* sp. YSU contains a single plasmid encoded mercury resistance operon (Figure 2a). The first *S. maltophilia* 02 mercury resistance operon (S02-*mer1*) contains *merR*, *merT*, *merP*, *merA*, *merD* and *merE*. The second operon (S02-*mer2*) contains *merR*, *merT*, *merP*, *merC* and *merA*. The *Enterobacter* sp. YSU mercury resistance operon (YSU-*mer*) contains *merR*, *merT*, *merP*, *merC*, *merA*, *merD* and *merE*.

To determine if these operons are functional, they were PCR amplified and cloned into plasmid, pSC-A-amp/kan, using the StrataClone PCR Cloning Kit to create the plasmids, pS02-*mer1*, pS02-*mer2* and YSU-*mer*, respectively. The complete S02-*mer1* and S02-*mer2* operons were cloned, and most of the YSU-*mer* operon was cloned. Only part of *merE* was missing. Then, *S. maltophilia* 02 and *Enterobacter* sp. YSU colonies were spotted onto R3A-tris agar plates along with *E. coli* containing one of the three recombinant plasmids or pSC-A-amp/kan as a control. This master plate was replica plated onto R3A-tris agar plates containing different concentrations of HgCl_2_. *S. maltophilia* 02, *Enterobacter* sp. YSU and *E. coli* containing all three recombinant plasmids grew at HgCl_2_ concentrations of 70 µM and below but not at 80 to 100 µM. However, *E. coli* containing the pSC-A-amp/kan vector grew at 0 and 10 µM HgCl_2_ but at none of the other concentrations. Since the inserts increased the HgCl_2_ MICS, all three operons conferred resistance to mercury in *E. coli*.

All three *mer* operons appear to encode MerR switches that sense mercury (Figure 2b). MEGA software alignment of the MerR amino acid residue sequences with Tn*21* MerR shows that all three have characteristic DNA binding domains, coupling domains and metal binding domains with three metal binding cysteine residues, a dimerization helix, a metal binding loop and a two-turn α-helix. MerR from the S02-*mer1* operon (QPX92366) appears to be closely related to MerR from Tn*21*, and MerR from the S02-*mer2* operon (QPX94025) appears to be more closely related to MerR from *Pseudomonas aeruginosa* (ALY42794.1). MerR from the YSU-*mer* operon appears to be more closely related to MerR from a plasmid of *Enterobacter cloacae* strain DH4 (QDS02919.1). MerR from YSU-*mer* was not annotated because it appears to be located on a plasmid that is at least 100 kb in size. Since the next-generation sequencing method used was not designed for plasmids, the sequence data for this plasmid was not uploaded to the NCBI database.

MEGA software alignment of the promoter regions of each operon shows that the −35 and −10 promoter regions are almost identical to those of the Tn*21* promoter [41] with slight variations in the −10 promoter regions of S02-*mer2* and YSU-*mer* (Figure 2c). They all contain a non-optimal 19 bp spacer between the −10 and −35 RNA polymerase binding sites. Like Tn*21*, the putative MerR binding-sites (operators) are all inverted repeats for S02-*mer1* and S02-*mer2*. The inverted repeat for MerR in YSU-*mer* contains one mismatch. Overall, the similarities in the MerR protein structures and promoters in the *S. maltophilia* 02 and *Enterobacter* sp. YSU operons suggest that they could be coupled with a reporter gene to form effective whole-cell Hg(II) biosensors.

### 3.2. Potential Enterobacter sp. YSU Zinc Sensor Switch, ZntR

**Figure 3:**
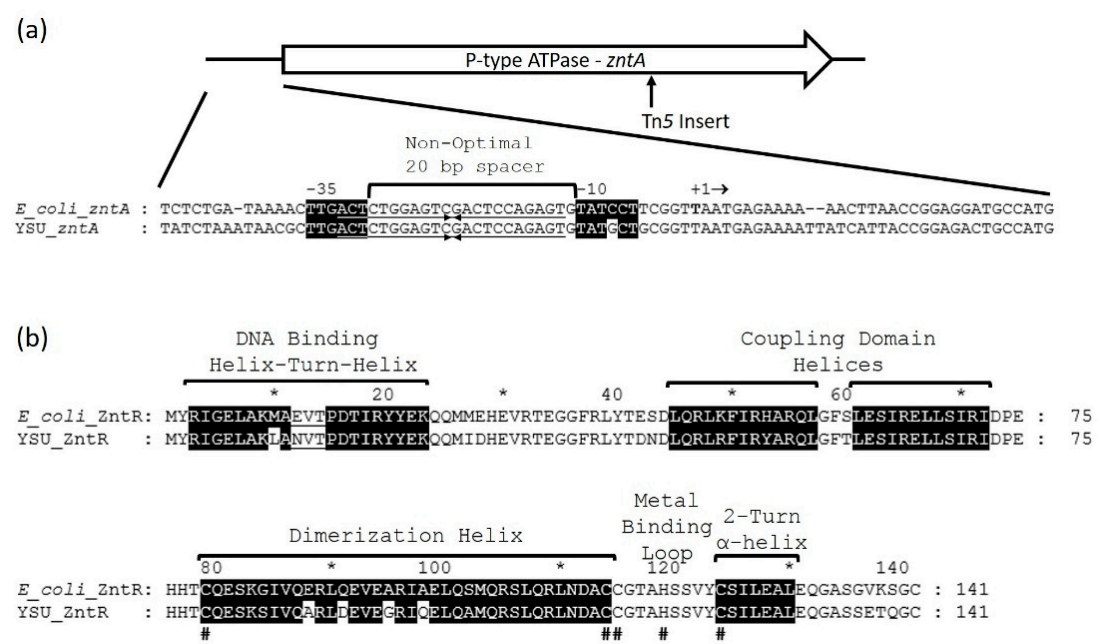
The *Enterobacter* sp. YSU zinc resistance (*znt*) operon and the ZntR activator/repressor switch. (**a**) The *Enterobacter* sp. YSU *zntA* gene and its predicted promoter region using the *E. coli zntA* promoter region. The gene for *zntR* is not adjacent to *zntA* and is located on a different region of the chromosome. An arrow indicates the Tn*5* transposon insertion sites in *zntA*. It inserted in almost the same site for both mutants. The promoter region contains a non-optimal 20 bp spacer region and a ZntR binding site (underlined arrows) between the predicted −10 and −35 regions. Identical nucleotides in the −10 and −35 regions are highlighted in black. The last three base pairs, ATG, on the far right marks the beginning of ZntA translation. (**b**) Prediction of the *Enterobacter* sp. YSU ZntR secondary structure using the *E. coli* ZntR amino acid residue primary sequence. Like MerR, the ZntR helix-turn-helix domain binds to the operator region of the promoter in the DNA. The coupling domain links the DNA binding domain to the metal binding domain. The metal binding domain consists of a dimerization helix, a metal binding loop and a 2-turn α-helix. The amino acids residues that are highlighted in black are identical to amino acid residues in the helices of *E. coli* ZntR. The # below the sequences designates cysteine and histidine amino acid residues which bind to Zn(II). The * and numbers above the ZntR primary sequences denote the position of every 10 amino acid residues.

Transposon mutagenesis and BLAST analysis of the *Enterobacter* sp. YSU genome was used to identify an *Enterobacter* sp. YSU zinc resistance gene and a ZntR protein switch that activates expression in the presence of Zn(II). The EZ-Tn*5* <R6K*γori*/KAN-2> transposome consists of a transposon DNA fragment containing an R6K*γ* replication origin and a kanamycin resistance gene flanked by mosaic ends for transposase enzyme binding [38,39,40]. The transposome is the transposon with transposase bound to the mosaic ends. When the transposome was introduced into *Enterobacter* sp. YSU, it randomly inserted the transposon into the bacterium’s genome, producing thousands of kanamycin-resistant colonies. After screening 1760 colonies on R3A-tris agar medium containing 1 mM ZnCl_2_, two zinc sensitive mutants, F24 and EI24, were identified. Purified genomic DNA from these mutants was digested with enzymes that do not cut the transposon, ligated and transformed into *E. coli*. The resulting transformants possessed a circular recombinant plasmid containing the transposon flanked by the interrupted genomic region. DNA sequencing and BLAST analysis against the *Enterobacter* sp. YSU genome showed that the transposon inserted itself into a gene that matched to the Zn(II)/Cd(II)/Pb(II) translocating P-type ATPase, ZntA (QPX99562), which confers resistance by pumping metal out of the cell (Figure 3a) [42].

The metal sensitive phenotype of F24 and EI24 was tested by replica plating. *Enterobacter* sp. YSU and each mutant was spotted in triplicate on R3A-tris agar plates without metal, grown overnight at 30 °C and replica plated onto R3A-tris agar plates containing different concentrations of ZnCl_2_, CdCl_2_ or Pb(NO_3_)_2_. After growing the plates overnight, the Zn(II) MIC for *Enterobacter* sp. YSU was between 4 and 5 mM, compared to between 0.2 and 0.3 mM for both mutants. The Cd(II) MIC for *Enterobacter* sp. YSU was between 0.2 and 0.3 mM compared to 0.01 and 0.05 mM for both mutants. The Pb(II) MIC for *Enterobacter* sp. YSU and the mutants was between 5 and 6 mM. Since the mutations decreased the Zn(II) and Cd(II) MICs but not the Pb(II) MIC, the *Enterobacter* sp. YSU *zntA* gene was required for zinc and cadmium resistance but not for lead resistance.

The *Enterobacter* sp. YSU ZntR protein (QPX99701) appears to be a MerR family switch that regulates the expression of the *zntA* gene [43]. Like *E. coli*, the gene for *zntR* is not adjacent to *zntA* and is located in a different region of the *Enterobacter* sp. YSU genome. The ZntR protein has the same structure as other MerR family protein switches [17]. MEGA software alignment with the *E. coli* ZntR protein (P0ACS5.1) shows that the *Enterobacter* sp. YSU ZntR protein contains a DNA binding domain, a coupling domain and a metal binding domain with a dimerization helix, a metal binding loop and a two-turn α-helix (Figure 3b). Two ZntR polypeptides exist as a dimer with four cysteine amino acid residues, C79, C114, C115 and C124, and one histidine amino acid residue, H119, forming one of two zinc binding domains, in which each domain binds to two Zn(II) cations. The dimerization helices form an antiparallel coiled coil so that C114, C115, H119 and C124 are supplied by one monomer and C79 is supplied by the other monomer.

The promoter for *zntA* in *Enterobacter* sp. YSU is similar to the promoter for *zntA* in *E. coli* (Figure 3a). Their −35 and −10 RNA polymerase binding sites are almost identical and instead of containing a 19 base pair non-optimal spacer between their −35 and −10 regions, they contain a 20 base pair non-optimal spacer [43]. Their ZntR binding, inverted repeats (operators) are identical. From these results, replacing the *zntA* gene with a reporter gene could produce a potential whole-cell biosensor for Zn(II) and Cd(II) cations.

### 3.3. Potential Enterobacter sp. YSU Copper Sensor Switch, CueR

Transposon mutagenesis of *Enterobacter* sp. YSU also yielded a copper sensitive mutant named LF88. In this case, BLAST analysis against the *Enterobacter* sp. YSU genome showed that the transposon inserted itself into a gene for a copper-exporting P-type ATPase, CopA (QPX98190) [44]. Replica plating showed that the MIC was between 2 and 3 mM CuSO_4_ for *Enterobacter* sp. YSU and between 0.1 and 0.5 mM CuSO_4_ for the LF88 mutant. Since *E. coli copA* expression is also induced in response to gold [45], the gold MIC was determined for *Enterobacter* sp. YSU and LF88. The MIC for *Enterobacter* sp. YSU and the LF88 mutant were both between 40 and 50 µM HAuCl_4_·3H_2_O. Since the wild type and mutant demonstrated no difference in gold resistance, the *Enterobacter* sp. YSU *copA* gene only appears to confer resistance to copper.

**Figure 4:**
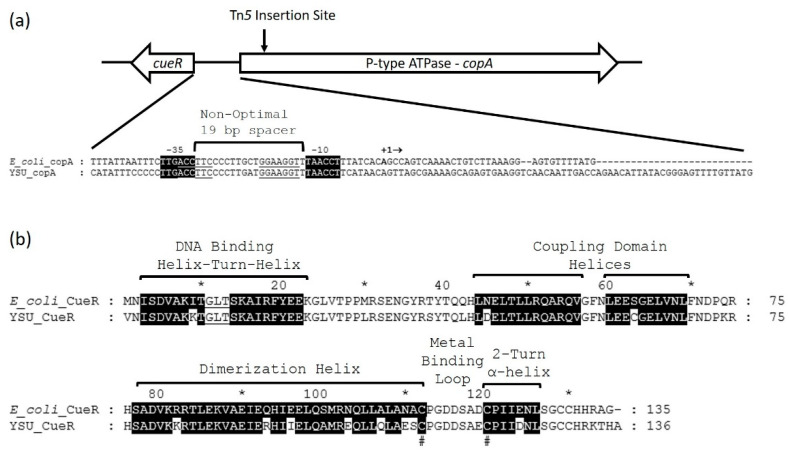
The *Enterobacter* sp. YSU copper resistance operon and the CueR activator/repressor switch. (**a**) The *Enterobacter* sp. YSU *copA* gene and its predicted promoter region using the *E. coli copA* promoter region. The gene for *cueR* is adjacent to *copA* in *Enterobacter* sp. YSU but not in *E. coli*. An arrow indicates the Tn*5* transposon insertion site in *copA*. The promoter region contains a non-optimal 19 bp spacer region and a CueR binding site (underlined) between the predicted −10 and −35 regions. Identical nucleotides in the −10 and −35 regions are highlighted in black. The first three base pairs, CAT, on the far left of the YSU_copA sequence marks the beginning of CueR translation in *Enterobacter* sp. YSU. The last three base pairs, ATG, on the far right marks the beginning of CopA translation in both strains. (**b**) Prediction of the *Enterobacter* sp. YSU CueR secondary structure using the *E. coli* CueR amino acid residue primary sequence. Like MerR, the CueR helix-turn-helix domain binds to the operator region of the promoter in the DNA. The coupling domain links the DNA binding domain to the metal binding domain. The metal binding domain consists of a dimerization helix, metal binding loop and 2-turn α-helix. The amino acids residues that are highlighted in black are identical to amino acid residues in the helices of *E. coli* CueR. The # below the sequences designates cysteine amino acid residues which bind to Cu(I). The * and numbers above the CueR primary sequences denote the position of every 10 amino acid residues.

The *Enterobacter* sp. YSU CueR protein (QPX98189) appears to be a MerR family protein switch that regulates the expression of the *copA* gene [46]. The *cueR* gene is adjacent to *copA* and transcribed in the opposite direction in *Enterobacter* sp. YSU (Figure 4a). In *E. coli*, it is located in a different region of the chromosome. MEGA Software alignment of the *E. coli* CueR (WP_135301207.1) amino acid residue sequence with *Enterobacter* sp. YSU CueR suggests that the *Enterobacter* sp. YSU CueR protein contains similar MerR family DNA binding, coupling and metal binding domains (Figure 4b) [17]. After the cell reduces Cu(II) to Cu(I), CueR binds to Cu(I) using two cysteine amino acid residues, Cys112 and Cys120. The dimerization helices from two monomers are arranged in an antiparallel orientation to form a coiled coil in a homodimer, but the cysteine amino acid residue at position 77 is replaced by a serine amino acid residue, which is not involved in Cu(I) binding.

The *copA* promoter regions for *E. coli* and *Enterobacter* sp. YSU are similar (Figure 4a) [47]. They share identical −35 and −10 RNA polymerase binding regions, and inverted repeat CueR binding sites. Their non-optimal 19 base pair spacers differ by one base pair, and their transcription ATG start sites are different. Replacing the *copA* gene in *Enterobacter* sp. YSU with a reporter gene could produce a potential whole-cell Cu(II)/Cu(I) cation biosensor.

### 3.4. Potential S. maltophilia 02 Gold and Copper Sensor Switch, CueR

**Figure 5:**
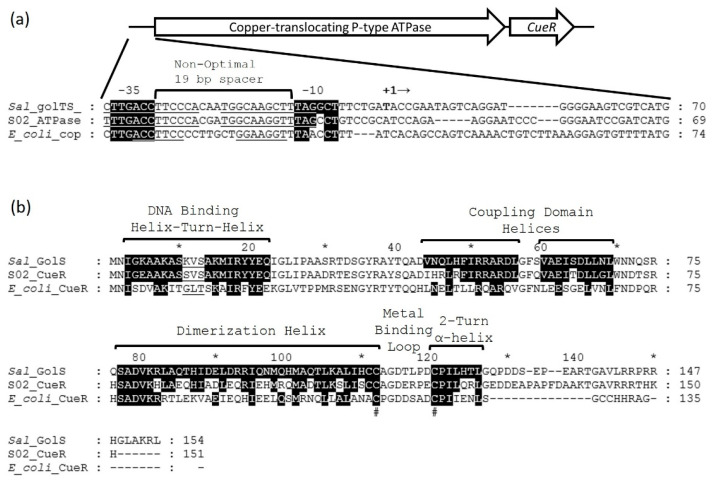
The *S. maltophilia* 02 copper translocating P-type ATPase gene and CueR activator/repressor switch. (**a**) The *S. maltophilia* 02 copper translocating P-type ATPase gene and its predicted promoter region using the *S. enterica golT*, and the *E. coli copA* promoter regions. The gene for *cueR* is adjacent to and transcribed after the ATPase gene in *S. maltophilia* 02. The promoter region contains a non-optimal 19 bp spacer region and a CueR binding site (underlined) between the predicted −10 and −35 regions. Identical nucleotides in the −10 and −35 regions are highlighted in black. The last three base pairs, ATG, on the far right marks the beginning of ATPase translation. (**b**) Prediction of the *S. maltophilia* 02 CueR secondary structure using the *S. enterica* GolS and the *E. coli* CueR amino acid residue primary sequences. Like MerR, the CueR helix-turn-helix domain binds to the operator region of the promoter in the DNA. The coupling domain links the DNA binding domain to the metal binding domain. The metal binding domain consists of a dimerization helix, metal binding loop and 2-turn α-helix. The amino acids residues that are highlighted in black are identical to amino acid residues in the helices of *E. coli* CueR. The # below the sequences designates cysteine amino acid residues which bind to Au(I) and Cu(I). The * and numbers above the Gols and CueR primary sequences denote the position of every 10 amino acid residues.

*S. maltophilia* 02 is resistant to HAuCl_4_·3H_2_O [29]. A BLAST search using the *Salmonella enterica* gold resistance protein GolT (QAX67042), a gold translocating P-type ATPase [48], against the *S. maltophilia* 02 genome identified a copper-translocating P-type ATPase (QPX92549) that was 83% similar to GolT. The gene for the *S. maltophilia* P-type ATPase along with the adjacent gene for a MerR family protein, CueR (QPX92548), was PCR amplified (Figure 5a) and cloned into the vector plasmid, pSC-A-amp/kan. Replica plating onto different concentrations of gold and copper measured levels of resistance. The MIC for *S. maltophilia* 02 was between 2 and 3 mM for CuSO_4_ and between 30 and 40 µM for HAuCl_4_·3H_2_O. The MIC for *E. coli* containing the pSC-A-amp/kan vector and the vector with the ATPase/*cueR* insert was between 0.5 and 1 mM for CuSO_4_ and between 10 and 20 µM for HAuCl_4_·3H_2_O. Since the insert did not increase the MICs for either metal, it did not confer resistance to copper or gold when introduced into *E. coli*.

**Figure 6:**
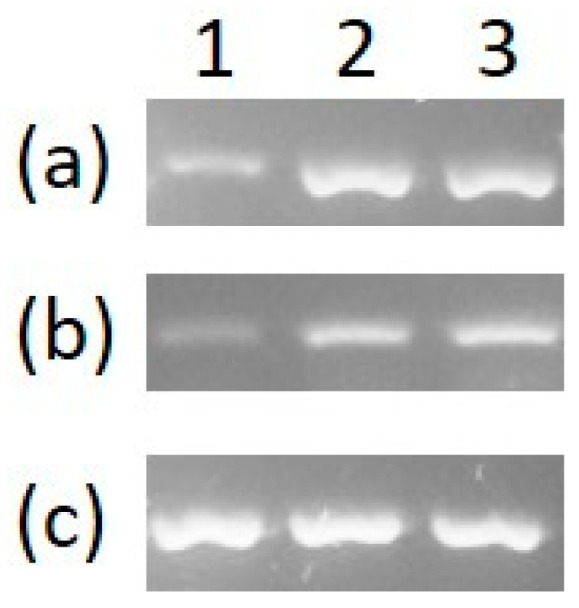
Expression of the *S. maltophilia* 02 copper translocating P-type ATPase gene and *cueR*. During exponential growth, *S. maltophilia* 02 was exposed to 1 mM CuSO_4_, 200 µM HAuCl_4_·3H_2_O or no metal in three separate cultures. One hour after exposure, total RNA was purified from each culture and converted to cDNA using reverse transcriptase. The cDNA was then PCR amplified using primers specific for the copper P-type ATPase translocation gene, the *cueR* gene and the glyceraldehyde-3-phosphate dehydrogenase (GAPDH) gene. The PCR reactions were separated using a 2% agarose gel. (**a**) The copper P-type ATPase translocation gene, (**b**) *cueR* and (**c**) GAPDH. (Lane 1) no metal, (Lane 2) copper and (Lane 3) gold. The more intense bands for copper and gold suggest that expression of the ATPase and *cueR* genes increases in response to copper and gold.

Reverse transcriptase polymerase chain reaction (RT-PCR) was performed to see if the genes for the *S. maltophilia* 02 P-Type ATPase and *cueR* are expressed in response to copper and gold. Cultures of *S. maltophilia* were grown to early mid-log phase and exposed to 200 µM HAuCl_4_·3H_2_O, 1 mM CuSO_4_ or an equal volume of water. After 1 h of exposure, cells were harvested for RNA purification, and 93 ng of RNA was converted to cDNA using reverse transcriptase. PCR reactions were performed using the cDNA as a template and primers for the GAPDH gene as a housekeeping control, for the ATPase gene and for the *cueR* gene. Agarose gel electrophoresis showed that the expression levels for both the ATPase gene (Figure 6a) and *cueR* (Figure 6b) increased in response to copper and gold because the intensity of the DNA bands in lanes 2 (copper treated) and 3 (gold treated) are greater than the intensity of the DNA bands in lane 1 (water control). The equal intensity in DNA bands for the GAPDH housekeeping gene (Figure 6c) under all conditions confirmed that equal amounts of RNA were used for all conditions. Thus, it appears that the *S. maltophilia* 02 CueR switch protein regulates expression of both the ATPase gene and itself, repressing expression in the absence of metal and inducing it in the presence of copper and gold.

The *S. enterica* protein, GolS (QAX67041), is a MerR family protein switch, which activates the expression of *golTS* in the presence of HAuCl_4_·3H_2_O [49]. MEGA software alignment of the GolS protein with the *S. maltophilia* 02 and the *E. coli* CueR proteins show that they possess the typical MerR family DNA binding, coupling and metal binding domains (Figure 5b) [48,50]. The *S. maltophilia* 02 CueR protein is more related to GolS than it is to *E. coli* CueR in the DNA binding helix, coupling domain helix and 2-turn α-helix but less similar in the dimerization helix. Inside the cell, Au(III) is reduced to Au(I). Then, similar to CueR binding to Cu(I), GolS binds to Au(I) using two cysteine amino acid residues at positions Cys 112 and Cys120.

The *S. maltophilia* P-type ATPase gene promoter region is also similar to the *golTS* promoter region [51,52]. The −35 and −10 promoter regions of the *S. maltophilia* 02 P-type ATPase are almost identical to the −35 and −10 *golTS* promoter regions. They also both have a non-optimal 19 bp spacer and almost identical inverted repeats for DNA binding by CueR. From these results, replacing the *S. maltophilia* 02 P-type ATPase gene with a reporter gene and leaving the *cueR* gene intact could produce a potential Au(I)/Au(III) and Cu(I)/Cu(II) cation biosensor.

### 3.5. Other Reporter Genes for MerR Family Biosensors

In addition to the gene for β-galactosidase, there are other genes that may serve as reporters in whole-cell biosensors [1,2,3,25,53,54,55,56]. Several investigators used bioluminescence to detect metals. Luciferase is a protein that gives off light. For example, Selifonova et al. used a luciferase reporter to measure Hg(NO_3_)_2_ at nanomolar and micromolar concentrations [57] and were able to increase the sensitivity to picomolar concentrations by decreasing the number of cells in the assay [58]. Light emissions were measured using a scintillation counter with linear readings ranging between 10^5^ and 10^7^ quanta/sec/mL. Brocklehurst et al. replaced the *zntA* gene in *E. coli* with the luciferase genes and were able to detect Zn(II) concentrations between 0.1 µM and 1.1 mM using a luminometer to detect light emissions [43]. Other investigators used the gene for the green fluorescence protein (GFP) as a reporter. When exposed to ultraviolet (UV) light, GFP fluoresces green. Pang et al. replaced the *copA* gene with the GFP gene in *E. coli* and detected CuSO_4_ concentrations between 0.39 and 78.68 μM [59]. Fluorescence was measured using a fluorescence spectrometer with linear readings ranging between 50 and 500 absolute fluorescence units (AFU). Özyurt et al. linked MerR to the enhanced yellow fluorescent protein (EYFP) so that EYFP was inactive when MerR was not bound to Hg(II) and became active due to conformational changes when MerR bound Hg(II) [60]. The purified chimeric protein detected Hg(II) concentrations between 0.5 and 40 nM.

### 3.6. Improving the Sensitivity and Specificity of MerR Family Biosensors

Sensitivity can be improved if the resistance genes, such as *merA*, *zntA*, *copA* and *golT*, are deleted from the host’s genome. When they are intact, they remove the metal and keep the concentration inside the cell low. The MerR family switch then requires more metal to activate the reporter gene, decreasing the sensitivity of the biosensor. Pang et al. removed the *copA* gene from *E. coli* to improve the sensitivity of copper detection [59]. All MerR family metal biosensors require deletion of the original resistance genes, but keeping their promoters. This problem may also be solved by constructing a recombinant plasmid which contains the switch protein gene along with its target promoter which controls the expression of a reporter gene. Then, introducing it into a bacterial strain that lacks the resistance genes will improve the sensitivity of the assay.

Metal specificity is important for detecting specific metals without interference by other metals. A previous random mutagenesis study of MerR to identify mutants that recognized Cd(II) instead of Hg(II) mainly resulted in constitutive expression [61]. Mutants that did induce expression in response to Cd(II) demonstrated some expression in the absence of metal and even higher levels of expression in the presence of Cd(II) and Hg(II). Recently, Hakkila et al. successfully mutagenized *merR* so that it will selectively respond to Cd(II) [62]. Most of the modifications were in the second coupling domain helix, at each end of the dimerization helix, in the metal binding loop and in the 2-turn α-helix. Kim et al. mutated the metal binding loop of ZntR so that it induced expression in response to Cd(II), Hg(II) and Pb(II) [63]. Recently, Ibáñez et al. showed that the metal binding loop is important for metal specificity in *Salmonella* [48,64]. They removed the metal binding loop region in GolS and replaced it with the metal binding loop region of CueR. The chimeric protein responded to both gold and copper when the wild type only responded to gold. In addition, changing serine at position 77 to cysteine in the N-terminal end of the dimerization helix of GolS and CueR maintained their response to gold and copper, and increased their response to the divalent cations, mercury, zinc, cadmium, and lead [64].

MerR family protein DNA binding domains may also be exchanged to change the specificity of the biosensor. Brockelhurst et al. swapped out the DNA binding domain of ZntR with the DNA binding region of MerR [43]. This chimeric protein strongly induced expression from the *merTPCADE* promoter in response to Zn(II) and demonstrated a weaker to response to Hg(II). Humbert et al. performed a similar experiment with CueR and GolS using a *lacZ* reporter [51]. They exchanged DNA binding domains between these two proteins so that the CueR chimeric protein induced expression from a gold resistance promoter in the presence of copper and gold, and the GolS chimeric protein induced expression from a copper resistance promoter in the presence of copper and gold. Thus, specificity can be modified by swapping domains or modifying the amino acid residue sequence in the MerR family protein of interest.

There are several advantages to using whole-cell biosensors [65,66]. Bacterial biosensors cells are inexpensive, easy to grow and can be produced in high amounts in a short period of time. This allows for the possibility of performing high-throughput assays to analyze many samples at a time [67]. The equipment that detects reporter gene signals are less expensive, requires less training and tends to be more portable than other metal analyzing instruments. Biosensor assays also save time because sample processing before analysis is minimal compared to other analytical techniques. In addition, whole-cell biosensors with MerR family switch proteins that have different levels of specificities can be combined. A low specificity switch protein which activates reporter gene expression in response to multiple metals can be used to detect overall metal contamination. If there are a large number of samples to analyze, those that do not contain metal can be eliminated. Then, whole-cell biosensors with more selective switch proteins can be used to detect specific metals. Finally, whole-cell biosensors only detect bioavailable metals [57]. Other methods detect both the bioavailable and the inert presence of metal, making it difficult to accurately assess the safety of an environmental sample. Thus, whole-cell biosensors provide an easy and inexpensive way to determine the metal content of a sample, and the results can be further refined using more precise analytical techniques.

## 4. Conclusions

*S. maltophilia* 02 and *Enterobacter* sp. YSU have the potential to be used as whole-cell biosensors for the detection of metals such as mercury, zinc, copper and gold. We hypothesized that these multimetal resistant bacterial strains would use MerR family activator/repressor protein switches to regulate metal resistance operons, and sequencing of their genomes supported this prediction. Gene cloning, transposon mutagenesis, MIC experiments and RT-PCR suggested that *S. maltophilia* 02 contains genes for MerR and CueR, which regulate operons for mercury, gold and copper resistance, respectively. They also suggested that *Enterobacter* sp. YSU contains genes for MerR, ZntR and CueR, which regulate operons for mercury, zinc and copper resistance, respectively. Replacing the resistance genes with reporter genes such as, β-galactosidase, luciferase or GFP can make them potential biosensors for these metals. Each MerR family protein has a DNA binding domain, coupling domain, and a metal binding domain. Modifying regions in the metal-binding domain or swapping metal binding or DNA binding domains between MerR family proteins can enhance the sensitivity and specificity for metal detection. To use the MerR family proteins from *S. maltophila* 02 and *Enterobacter* sp. YSU as whole-cell biosensors, their genes will need to be modified.
